# Occurrence of *Terranova* larval types (Nematoda: Anisakidae) in Australian marine fish with comments on their specific identities

**DOI:** 10.7717/peerj.1722

**Published:** 2016-03-15

**Authors:** Shokoofeh Shamsi, Jaydipbhai Suthar

**Affiliations:** School of Animal and Veterinary Sciences, Charles Sturt University, Wagga Wagga, NSW, Australia

**Keywords:** Anisakidae, *Terranova*, Taxonomy

## Abstract

Pseudoterranovosis is a well-known human disease caused by anisakid larvae belonging to the genus *Pseudoterranova*. Human infection occurs after consuming infected fish. Hence the presence of *Pseudoterranova* larvae in the flesh of the fish can cause serious losses and problems for the seafood, fishing and fisheries industries. The accurate identification of *Pseudoterranova* larvae in fish is important, but challenging because the larval stages of a number of different genera, including *Pseudoterranova*, *Terranova* and *Pulchrascaris*, look similar and cannot be differentiated from each other using morphological criteria, hence they are all referred to as *Terranova* larval type. Given that *Terranova* larval types in seafood are not necessarily *Pseudoterranova* and may not be dangerous, the aim of the present study was to investigate the occurrence of *Terranova* larval types in Australian marine fish and to determine their specific identity. A total of 137 fish belonging to 45 species were examined. *Terranova* larval types were found in 13 species, some of which were popular edible fish in Australia. The sequences of the first and second internal transcribed spacers (ITS-1 and ITS-2 respectively) of the *Terranova* larvae in the present study showed a high degree of similarity suggesting that they all belong to the same species. Due to the lack of a comparable sequence data of a well identified adult in the GenBank database the specific identity of *Terranova* larval type in the present study remains unknown. The sequence of the ITS regions of the *Terranova* larval type in the present study and those of *Pseudoterranova* spp. available in GenBank are significantly different, suggesting that larvae found in the present study do not belong to the genus *Pseudoterranova*, which is zoonotic. This study does not rule out the presence of *Pseudoterranova* larvae in Australian fish as *Pseudoterranova decipiens E* has been reported in adult form from seals in Antarctica and it is known that they have seasonal presence in Australian southern coasts. The genetic distinction of *Terranova* larval type in the present study from *Pseudoterranova* spp. along with the presence of more species of elasmobranchs in Australian waters (definitive hosts of *Terranova* spp. and *Pulchrascaris* spp.) than seals (definitive hosts of *Pseudoterranova* spp.) suggest that *Terranova* larval type in the present study belong to either genus *Terranova* or *Pulchrascaris*, which are not known to cause disease in humans. The present study provides essential information that could be helpful to identify Australian *Terranova* larval types in future studies. Examination and characterisation of further specimens, especially adults of *Terranova* and *Pulchrascaris*, is necessary to fully elucidate the identity of these larvae.

## Introduction

Psudoterranovosis (Hochberg & Hamer, 2010), the seafood borne parasitic disease, caused by larvae of *Pseudoterranova*, is another form of anisakidosis, that has caused concern to human beings. The disease is most common in the United States followed by Japan and Europe (Hochberg & Hamer, 2010). With the increased popularity of eating raw or slightly cooked seafood dishes, the number of cases have increased globally (Chai, Darwin Murrell & Lymbery, 2005). The symptoms of the disease vary and may include nausea, severe epigastric pain and other abdominal discomforts, “tingling throat syndrome” from a worm crawling in the upper esophagus or oropharynx, cough and vomiting up live or dead worms (Margolis, 1977). The life cycle of the *Pseudoterranova* spp. includes crustaceans and fish as their intermediate hosts and marine mammals as their definitive hosts (Anderson, 2000). Human infection occurs after eating infected seafood, therefore the presence of *Pseudoterranova* larvae in the flesh of fish can cause serious losses and problems for fish and fisheries industry across the world. For example, up to 36 worms per fish have been reported in cod populations from Norwegian waters (Jensen, Andersen & Desclers, 1994) or Icelandic cod fillets provided by the industry have been reported to be infected with 2.5–17.6 worms per kg fillet (Hafsteinsson & Rizvi, 1987). It has been estimated that detection and removal of the larvae thought to be *Pseudoterranova* from the flesh of Atlantic cod (*Gadus morhua*) and other demersal species, and the resultant downgrading and discard of product, cause an annual loss of $50 million in Atlantic Canada (McClelland, 2002). This implies the need for detection and accurate identification of these larvae in fish. One of the challenges in diagnosing of parasitic diseases is the specific identification of larval stages of parasites. Larval stages of nematodes cannot be identified reliably using morphological characters alone. This is a consequence of the small size of larval stages and the lack of a sufficient number of characteristic features (Shamsi, Gasser & Beveridge, 2011). Molecular approaches have gained prominence for accurate identification of anisakids, irrespective of developmental stage and sex of the parasite, and for establishing systematic relationships (e.g., Orecchia et al., 1986). Several studies showed that ITS-1 and ITS-2 are useful genetic markers for specific identifications of nematodes irrespective of their developmental stage or sex and to study their life cycle (e.g., Shamsi, Gasser & Beveridge, 2011). However, this approach relies on presence of ITS sequences for well identified adults.

In several countries other than Australia, the ability to recognise and diagnose anisakidosis/pseudoterranovosis caused by these larvae has been improved, resulting in progress towards understanding its epidemiology and clinical manifestations of the disease. In Australia, however, little is known about the disease, the causative agent and its epidemiology. Australia is an increasingly multicultural country where seafood prepared in all its forms is very popular. A confirmed case of human anisakidosis was published recently by Shamsi & Butcher (2011) and several unpublished cases are on record (Shamsi, 2014). Therefore, there has been an increasing awareness of anisakidosis in humans and the presence of anisakid larval types in marine fish in Australia (Shamsi, 2014).

A review of the literature shows that *Terranova* larval types have been reported quite often in Australian marine fish (e.g., Cannon, 1977; Doupe et al., 2003; Lester, Barnes & Habib, 1985; Moore et al., 2011) but there is no information on the specific identity of *Terranova* larval types reported in Australia. The dilemma with *Terranova* larval types is that it could belong to any of three genera of anisakid nematodes, including *Terranova, *Pulchrascaris** or *Pseudoterranova,* whose adult stages have been reported from Australian waters. Members of *Terranova* and *Pulchrascaris* become adult in elasmobranchs and are not known to cause harm to humans whereas *Pseudoterranova* spp. become adult in marine mammals and there are numerous publications about their pathogenicity and human health impacts. The larval stages of all these genera, i.e., *Terranova*, *Pseudoterranova* and *Pulchrascaris* are morphologically very similar. The typical characteristic of these larvae is the location of the excretory pore at the anterior end of the nematode, presence of a ventriculus without an appendix and having an intestinal caecum (Deardorff, 1987; Gibson & Colin, 1982). Therefore, distinction between larval stages of these genera based solely on morphology can be challenging. With recent increasing awareness about the presence of anisakid larvae in Australian fish as well as the presence of human cases in the country, knowing the specific identity of *Terranova* larval types becomes very important. In the last decade, molecular tools have provided the opportunity for specific identification of larval stages of parasites and there have been several works in the Americas, European countries and Antarctica on specific identification of *Terranova* larval types (Arizono et al., 2011; Paggi et al., 1991). Therefore, the aim of the present study is to employ a combined molecular and morphological approach to investigate the occurrence of *Terranova* larval types in Australian marine fish and to determine their specific identity.

## Materials and Methods

### Parasite collection

A total of 137 fish belonging to 45 species, *Abudefduf whitleyi* (*n* = 2), *Aldrichetta forsteri* (*n* = 1), *Atherinomorus vaigiensis* (*n* = 1), *Caesio cuning* (*n* = 8), *Carangoides fulvoguttatus* (*n* = 1), *Caranx ignobilis* (*n* = 2), *C. melampygus* (*n* = 1), *Carcharias taurus* (*n* = 1), *Chaetodon aureofasciatus* (*n* = 1), *C. auriga* (*n* = 1), *C. flavirostris* (*n* = 2), *C. lineolatus* (*n* = 1), *C. melannotus* (*n* = 1), *Chaetodon sp* (*n* = 1), *Coryphaena hippurus* (*n* = 1), *Engraulis australis* (*n* = 2), *Epinephelus cyanopodus* (*n* = 1), *Grammatorcynus bicarinatus* (*n* = 3), *Haplophryne sp.* (*n* = 1), *Heniochus monoceros* (*n* = 2), *H. singularius* (*n* = 1), *Istiompax indica* (*n* = 3), *Kajikia audax* (*n* = 3), *Lutjanus argentimaculatus* (*n* = 2), *L. bohar* (*n* = 1), *L. carponotatus* (*n* = 4), *L. fulviflamma* (*n* = 1), *L. sebae* (*n* = 4), *Makaira mazara* (*n* = 3), *Mugil cephalus* (*n* =5), *Pastinachus sephen* (*n* = 1), *Platycephalus laevigatus* (*n* = 8), *Platycephalus sp.* (*n* = 2), *Pristipomoides multidens* (*n* = 3), *Rhombosolea tapirina* (*n* = 3), *Sardinops sagax neopilchardus* (*n* = 8), *Scomber australasicus* (*n* = 11), *Seriola hippos* (*n* = 2), *S. lalandi* (*n* = 17), *Siganus fuscescens* (*n* = 1), *S. punctatus* (*n* = 1), *Sillago flindersi* (*n* = 13), *Sphyraena novaehollandiae* (*n* = 4), *Taeniomembras microstomus* (*n* = 1), and *Thunnus albacares* (*n* = 1) were examined for infection with anisakid larval types. Fish were collected off Australian coasts, including Queensland, New South Wales, Victoria, South Australia and Western Australia. No fish were caught or killed for the purpose of this study. All fish were either already euthanized as part of other research projects or were bought from fishermen in various fish markets.

**Table 1:** Scientific name and specimens/accession number of taxa used to build phylogenetic trees in the present study.

Abbreviation	Scientific name	Specimen/Accession no.		Reference
		ITS-1	ITS-2	
A.brevispiculata	*Anisakis brevispiculata*	AY826719	AY826719	Nadler et al. (2005)
A.brevispiculata1	*Anisakis brevispiculata*	PSW4-1	PSW4-2	Shamsi, Gasser & Beveridge (2012)
A.pegreffii	*Anisakis pegreffii*	FN391850	FN556997	Shamsi, Gasser & Beveridge (2012)
A.pegreffii1	*Anisakis pegreffii*	FN391851	FN556998	Shamsi, Gasser & Beveridge (2012)
A.physeteris	*Anisakis physeteris*	AY826721	AY826721	Nadler et al. (2005)
A.physeteris1	*Anisakis physeteris*	AY603530	AY603530	Kijewska et al. (2008)
A.simplexC	*Anisakis simplex* C	FN391883	FN391884	Shamsi, Gasser & Beveridge (2012)
A.simplexs.s.	*Anisakis simplex sensu stricto*	AJ225065	AB196672	Abe, Ohya & Yanagiguchi (2005)
A.TMTP	Larva of *Anisakis* sp. (TMTP)	AY260555	AY260555	Pontes et al. (2005)
A.typica	*Anisakis typica*	AY826724	AY826724	Nadler et al. (2005)
A.typica1	*Anisakis typica*	FN391887	FN391889	Shamsi, Poupa & Justine (2015)
A.ziphidarum	*Anisakis ziphidarum*	AY826725	AY826725	Nadler et al. (2005)
C.bancrofti	*Contracaecum bancrofti*	EU839572	FM177883	Shamsi et al. (2009b)
C.bancrofti1	*Contracaecum bancrofti*	EU839573	FM177887	Shamsi et al. (2009b)
C.eudyptulae	*Contracaecum eudyptulae*	FM177531	FM177562	Shamsi et al. (2009b)
C.margolisi	*Contracaecum margolisi*	AY821750	AY821750	Nadler et al. (2005)
C.microcephalum	*Contracaecum microcephalum*	FM177524	FM177528	Shamsi et al. (2009b)
C.multipapillatum	*Contracaecum multipapillatum*	AM940056	AM940060	Shamsi et al. (2008)
C.ogmorhini	*Contracaecum ogmorhini sensu stricto*	FM177542	FM177547	Shamsi et al. (2009b)
C.osculatumA	*Contracaecum osculatum* A	AJ250410	AJ250419	Zhu et al. (2000)
C.osculatumB	*Contracaecum osculatum* B	AJ250411	AJ250420	Zhu et al. (2000)
C.osculatumbaicalensis	*Contracaecum osculatum baicalensis*	AJ250415	AJ250416	Zhu et al. (2000)
C.osculatumC	*Contracaecum osculatum* C	AJ250412	AJ250421	Zhu et al. (2000)
C.osculatumD	*Contracaecum osculatum* D	AJ250413	AJ250418	Zhu et al. (2000)
C.osculatumE	*Contracaecum osculatum* E	AJ250414	AJ250417	Zhu et al. (2000)
C.radiatum	*Contracaecum radiatum*	AY603529	AY603529	Kijewska et al. (2002)
C.rudolphiiA	*Contracaecum rudolphii* A	AJ634782	AY603535	Li et al. (2005)
C.rudolphiiB	*Contracaecum rudolphii* B	AJ634783	AJ634911	Li et al. (2005)
C.rudolphiiD	*Contracaecum rudolphii* D	FM210253	FM210267	Shamsi et al. (2009a)
C.rudolphiiD1	*Contracaecum rudolphii* D	FM210254	FM210265	Shamsi et al. (2009a)
C.rudolphiiE	*Contracaecum rudolphii* E	FM210257	FM210269	Shamsi et al. (2009a)
C.rudolphiiE1	*Contracaecum rudolphii* E	FM210258	FM210273	Shamsi et al. (2009a)
C.septentrionale	*Contracaecum septentrionale*	AJ634784	AJ634787	Li et al. (2005)
C.variegatum	*Contracaecum variegatum*	FM177531	FM177537	Shamsi et al. (2009b)
Contracaecumn.sp.	*Contracaecumpyripapillatum*	AM940062	AM940066	Shamsi et al. (2009b)
H.aduncum1	*Hysterothylacium aduncum*	AJ225068	AJ225069	Zhu et al., 1998)
H.aduncum2	*Hysterothylacium aduncum*	AB277826	AB277826	Umehara et al. (2008)
H.auctum	*Hysterothylacium auctum*	AF115571	AF115571	Szostakowska et al. (2001)
H.III	*Hysterothylacium* larval type III	FN811721	FN811678	Shamsi, Gasser & Beveridge (2013)
H.III-1	*Hysterothylacium* larval type III	FN811723	FN811681	Shamsi, Gasser & Beveridge (2013)
H.IVA	*Hysterothylacium* larval type IV Genotype A	FN811724	FN811690	Shamsi, Gasser & Beveridge (2013)
H.IVB	*Hysterothylacium* larval type IV Genotype B	FN811730	FN811682	Shamsi, Gasser & Beveridge (2013)
H.IVGA	*Hysterothylacium* larval type IV Genotype A	FN811729	FN811690	Shamsi, Gasser & Beveridge (2013)
H.IVGA1	*Hysterothylacium* larval type IV Genotype A	FN811729	FN811691	Shamsi, Gasser & Beveridge (2013)
H.IVGA2	*Hysterothylacium* larval type IV Genotype A	FN811729	FN811692	Shamsi, Gasser & Beveridge (2013)
H.IVGB	*Hysterothylacium* larval type IV Genotype B	FN811730	FN811683	Shamsi, Gasser & Beveridge (2013)
H.IVGB1	*Hysterothylacium* larval type IV Genotype B	FN811731	FN811684	Shamsi, Gasser & Beveridge (2013)
H.IVGB2	*Hysterothylacium* larval type IV Genotype B	FN811733	FN811685	Shamsi, Gasser & Beveridge (2013)
H.V	*Hysterothylacium* larval type V	FN811738	FN811699	Shamsi, Gasser & Beveridge (2013)
H.VI	*Hysterothylacium* larval type VI	FN811740	FN811701	Shamsi, Gasser & Beveridge (2013)
H.VII	*Hysterothylacium* larval type VII	FN811749	FN811709	Shamsi, Gasser & Beveridge (2013)
H.VIII	*Hysterothylacium* larval type VIII	FN811750	FN811710	Shamsi, Gasser & Beveridge (2013)
Heterakisgallinarum	*Heterakis gallinarum*	JQ995320	JQ995320	Jimenez et al. (2012)
P.azarasi	*Pseudoterranova azarasi*	AJ413973	AJ413974	Zhu et al. (2002)
P.bulbosa	*Pseudoterranova bulbosa*	AJ413970	AJ413971	Zhu et al. (2002)
P.cattani	*Pseudoterranova cattani*	AJ413982	AJ413984	Zhu et al. (2002)
P.decipiens	*Pseudoterranova decipiens*	AJ413979	AJ413980	Zhu et al. (2002)
P.decipiens1	*Pseudoterranova decipiens*	AJ413979	AJ413978	Zhu et al. (2002)
R.acus	*Raphidascaris acus*	AY603537	AY603537	Kijewska et al. (2008)
Terranovasp.	*Terranova* sp.	LN795828	LN795872	The present study
Terranovasp.1	*Terranova* sp.	LN795851	LN795871	The present study

Dead fish were cut open and first examined for presence of larval nematodes in the surface of the internal organs and also for gross pathology. Then the gastro-intestinal tract from mouth to anus was examined for the presence of nematodes. All nematodes found were washed in physiological saline and then preserved in 70% ethanol. A small piece of the mid-body of each nematode was excised for molecular study, and the rest of the nematode were used for microscopy.

### Morphological examination

The anterior and posterior parts of each nematode were cleared in lactophenol and examined under a light microscope. *Terranova* larvae were identified according to the identification key proposed by Cannon (1977) and were selected for description and further molecular analyses. Illustrations were made using a microscope equipped with camera lucida.

### Molecular study

Genomic DNA (gDNA) was isolated from all individual larvae identified morphologically as *Terranova* larval type, by sodium dodecyl-sulphate/proteinase K treatment, column- purified (Wizard™ DNA Clean-Up; Promega, Madison, WI, USA) and eluted into 45 µl of water. PCR was used to amplify the ITS-1 and ITS-2 regions using primer sets SS1: 5′-GTTTCCGTAGGTGAACCTGCG-3′ (forward) and NC13R: 5′-GCTGCGTT CTTCATCGAT-3′ (reverse) for the former and SS2: 5′-TTGCAGACACATTGAGCACT-3′ (forward) and NC2: 5′-TTAGTTTCTTTTCCTCCGCT-3′ (reverse) for the latter region, and cycling conditions, initial 94 °C/5′, then 94 °C/30″, 55 °C/40″, 72 °C/40″ × 30 cycles, 72 °C/5′ extension and 4 °C (Shamsi & Butcher, 2011). An aliquot (4 µl) of each amplicon was examined on a 1.5% w/v agarose gel, stained with GelRed™ and photographed using a gel documentation system.

Representative samples based on host species and geographical locations were selected for sequencing. Sequences were aligned using the computer program ClustalX (Thompson et al., 1997) and then adjusted manually. Polymorphic sites were designated using International Union of Pure and Applied Chemistry (IUPAC) codes. Pair-wise comparisons of sequence differences (*D*) were determined using the formula *D* = 1 − (*M*∕*L*), where *M* is the number of alignment positions at which the two sequences have a base in common, and *L* is the total number of alignment positions over which the two sequences are compared (Chilton, Gasser & Beveridge, 1995).

Phylogenetic analysis of the nucleotide sequence data for combined ITS-1 and ITS-2 regions were conducted in PAUP 4.0. Table 1 shows details of the taxa used to build phylogenetic trees. Two tree-building methods, neighbour-joining and maximum parsimony were employed for phylogenetic analysis. The outgroup employed was *Heterakis gallinarum* (Nematoda: Heteakoidea; GenBank accession numbers JQ995320 and JQ995320 for ITS-1 and ITS-2, respectively).

**Table 2:** Morphological description of *Terranova* larval type found in the present study. All measurements are given in millimetres. Mean measurements are given, followed by the range in parentheses.

Taxonomically important morphological character	Measurement/description
Body length	6.6 (3.0–9.0)
Body width	0.24 (0.18–0.28)
Tooth	Present
Lips morphology	Inconspicuous
Distance of nerve ring from anterior end	0.37 (0.22–0.72)
Location of excretory pore	At anterior end
Oesophagus length	0.88 (0.4–1.14)
Ratio of oesophagus length to body length	14.3 (9.5–26.5%)
Ventriculus length	0.38 (0.24–0.54)
Intestinal caecum length	0.71 (0.50–0.90)
Tail morphology	Strongly annulated, conical, tapering smoothly
Tail length	0.13 (0.12–0.14)
Ratio of tail length to body length	2.2% (1.3–4.0%)

## Results

**Figure 1:**
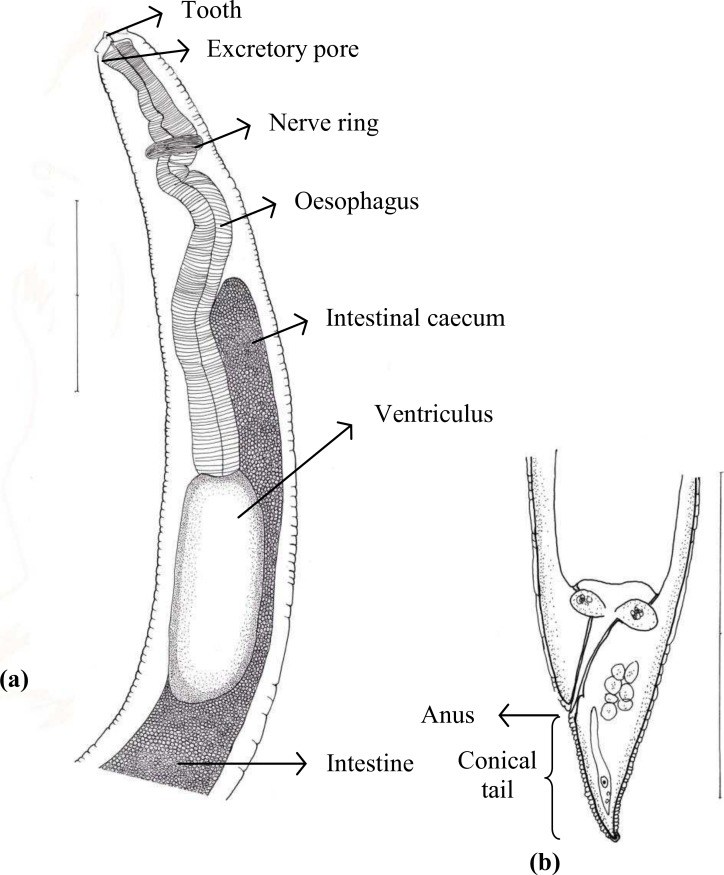
Diagram of *Terranova* larval type found in the present study indicating taxonomically important features (scale-bars = 0.3 mm).

Of 45 species of fish examined in the present study, third stage *Terranova* type larvae (*n* = 93) were identified as type II based on the presence of intestinal caecum and ventriculus, absence of developed labia and ventricular appendix, and location of the excretory pore being at the anterior end (Fig. 1). Morphological description of these larvae was summarized in Table 2. *Terranova* type larvae were found in 13 species of fish collected from North-Eastern, Eastern and south eastern coasts of Australia. Material morphologically examined were 10 larvae in good condition from *Caesio cuning* (*n* = 3), *Caranx ignobilis* (*n* = 2), *Grammatorcynus bicarinatus* (*n* = 1), *Lutjanus argentimaculatus* (*n* = 3) and *L. carponotatus* (*n* = 1) from Heron Island, Queensland.

**Table 3:** Taxa listed under genera *Terranova, Pulchrascaris* and *Pseudoterranova*.

Taxa	Host common name	Host scientific name	Location	Reference
***Terranova*** **Leiper & Atkinson, 1914**				
*T. amoyensis* Fang & Luo 2006	Red string ray	*Dasyatis akajei*	Taiwan Strait	Fang & Luo (2006)
*T. antarctica* (Leiper & Atkinson, 1914)^a^	Gummy shark	*Mustelus antarcticus*	Bay of Islands, New Zealand	Leiper & Atkinson (1914)
*T. brevicapitata* (Linton, 1901)	Tiger sharkk	*Galeocerdo cuvier*	Woods Hole, Massachusetts, USA	Deardorff (1987)
*T. caballeroi* Diaz-Ungria, 1967	Porcupine river stingray	*Potamotrygon hystrix*	Delta of the Orinoco River, Venezuela	Diaz-Ungria (1967)
*T. cephaloscyllii* (Yamaguti, 1941)	Blotchy swell shark	*Cephaloscyllium umbratile*	Nagasaki, Japan	Yamaguti (1941)
*T. circularis* (Linstow, 1907)	Common sawfish	*Pristis pristis*	Cameroon	Bruce, Adlard & Cannon (1994)
*T. crocodili* (Taylor, 1924)	West African crocodile Malayan crocodile	*Crocodylus sp Crocodylus johnstoni*	Ghana Northern Australia; Queensland; Malaya	Sprent, (1979)
*T. draschei* (Stossich, 1896)^b^	Arapaima	*Arapairna gigas*	Rivers of northern South America	Bruce, Adlard & Cannon (1994)
*T. galeocerdonis* (Thwaite, 1927)	Tiger shark Scalloped hammerhead Smooth hammerhead Blacktai reef shark	*Galeocerdo cuvier Sphyrna lewini S. zygaena Carcharinus amblyrhynchos*	Twynams Paar, Ceylon; South Australia and Queensland, Australia; Natal, northern Brazil.	Bruce, Adlard & Cannon (1994)
*T. ginglymostomae* Olsen, 1952^c^	Nurse shark Spotted wobbegong Zebra shark	*Ginglymostoma cirratum Orectolobus maculatusStegostoma fasciatum.*	Tortugas, Florida, USA; off Queensland, Australia	Bruce, Adlard & Cannon (1994)
*T. lanceolata* (Molin 1860)	Black caiman American alligator	*Melanosuchus niger Alligator mississippiensis*	Brazil	Sprent (1979)
*T. nidifex* (Linton, 1900)^d^	Tiger shark	*Galeocerdo tigrinus*	Woods Hole, Massachusetts, USA	Deardorff (1987)
*T. pristis* (Baylis & Daubney, 1922)	Largetooth sawfish Snaggletooth shark Wallago	*Pristis microdon (P. perotteti)Hemipristis elongatus Wallago attu*	Ulubaria, India; Balgal, Queensland, eastern Australia	Bruce, Adlard & Cannon (1994)
*T. petrovi* Mozgovoi, 1950^e^	Shark	*Raja longirostris*	Kamchatka, USSR	Bruce, Adlard & Cannon (1994)
*T. quadrata* (Linstow, 1904)	The saltwater crocodile	*Crocodilus porsus*	Belgrade	Mozgovoi (1950)
*T. rochalimai* (Pereira, 1935)^c^	Shark	Scientific name was not mentioned in the original description	Brazil	Mozgovoi (1950)
*T. scoliodontis* (Baylis, 1931)	Shark	*Scoliodon sp.*	Cleveland Bay, Townsville, Australia	Bruce, Adlard & Cannon (1994)
*T. secundum* (Chandler, 1935)^f^	Largehead hairtail	*Trichiurus lepturus.*	Galveston Bay, Texas, USA; La Paloma, Uraguay	Chandler (1935)
*T. serrata* (Drasche, 1896)^b^	Arapaima	*Arapaima gigas*	Rivers of northern South America	Bruce, Adlard & Cannon (1994)
*Terranova trichiuri* (Chandler, 1935)^g^	Indian threadfin	*Polydactylus indicus Trichiurus lepturus*	Galveston Bay, Texas, USA; Khulna, Pakistan	Bruce, Adlard & Cannon (1994)
***Pulchrascaris*** **Vicente and dos Santos, 1972**				
*P. caballeroi* Vicente and dos Santos, 1972	Angelshark	*Squatina squatina*^h^	Rio de Janeiro, Brazil	Bruce, Adlard & Cannon (1994)
*P. chiloscyllii* (Johnston and Mawson, 1951)	Brownbanded bambooshark Blacktip reef shark Gummy shark Scalloped hammerhead Smooth hammerhead Whitetip reef shark	*Chiloscyllium punctatumCarcharinus melanopterus Mustelus antarcticus Sphyrna lewini S. zygaena*Triaenenodon obesus	Halfway Island, Australia; Hawaii, Alabama, USA; South Africa	Bruce, Adlard & Cannon (1994)
*P. secunda* (Chandler, 1935)	Largehead hairtail	*Trichiurus lepturus.*	Galveston Bay, Texas, USA; La Paloma, Uraguay	Bruce, Adlard & Cannon (1994)
***Pseudoterranova*** **Mozgovoi, 1951**				
*Pseudoterranova azarasi* (Yamaguti & Arima, 1942)	Steller’s sea lion Californian sea lion Harbor seal Bearded seal	*Eumetopias jubatus Zalophus californianusPhoca vitulina richardsii Erignathus barbatus*	Japanese and Sakhalinese waters of the North Pacific Ocean	Mattiucci & Nascetti (2008)
*P. bulbosa* (Cobb, 1888)	Bearded seal	*Erignathus barbatus*	Barents and Norwegian Seas, the Canadian Atlantic and the Sea of Japan,	Mattiucci & Nascetti (2008)
*P*. *cattani* George-Nascimento and Urrutia, 2000	South American sea lion	*Otaria byronia*	South-East Pacific, Chilean coast	Mattiucci & Nascetti (2008)
*P. decipiens* (Krabbe, 1868) (sensu stricto)	Californian sea lion Harbor seal Harbor seal Grey seal Hooded seal Norhern elephant seal	*Zalophus californianus Phoca vitulina richardsii Phoca vituline Halichoerus grypus Cystophora cristataMirounga angustirostris*	North-East and North-West Atlantic	Mattiucci & Nascetti (2008)
*P. krabbei* Paggi, Mattiucci et al., 2000	Harbor seal Grey seal	*Phoca vituline Halichoerus grypus*	North-East Atlantic; Faeroe Islands	Mattiucci & Nascetti (2008)
*P. decipiens* E of Bullini et al., 1997	Antarctic Weddell seal	*Leptonychotes weddellii*	Antarctica	Mattiucci & Nascetti (2008)

**Notes.**

a The species has been described based on a single female and should be redescribed.

b Mozgovoi (1953) lists this species as *Terranova serrata* (Drasche 1884) while Bruce, Adlard & Cannon (1994) listed it as *Porrocaecurn draschei* (Stossich, 1896) and noted that there is some doubt as to which name has priority for this species.

c This taxon was considered as junior synonym of *T. galeocerdonis* by Tanzola & Sardella (2006).

d According to Johnston & Mawson (1945) *T. nidifex* may be identical to *T. galeocerdonis.*

e This taxon was regarded as species inquirenda by Gibson & Colin (1982).

f Now is known as *Pulchrascaris secunda* (Deardorff, 1987).

g This species was considered as a synonym of *T. secundum* (Chandler, 1935) by Olsen (1952).

h According to Deardorff (1987) this is a misidentification of host.

TM In the original description *Cação panan* was stated as type host which could not be assigned to any specific elsamobranch.

A total of 93 specimens from various fishes, including *Abudefduf whitleyi, Caesio cuning, Carangoides fulvoguttatus, Caranx ignobilis, Caranx melampygus, Epinephelus cyanopodus, Grammatorcynus bicarinatus, Lutjanus argentimaculatus, L. bohar, L. carponotatus* and *L. fulviflamma* and *Scomber australasicus* were subjected to PCR amplification. Based on the species of hosts and their geographical locations, 25 and 21 specimens were selected and sequenced for ITS-1 and ITS-2 respectively.

**Figure 2:**
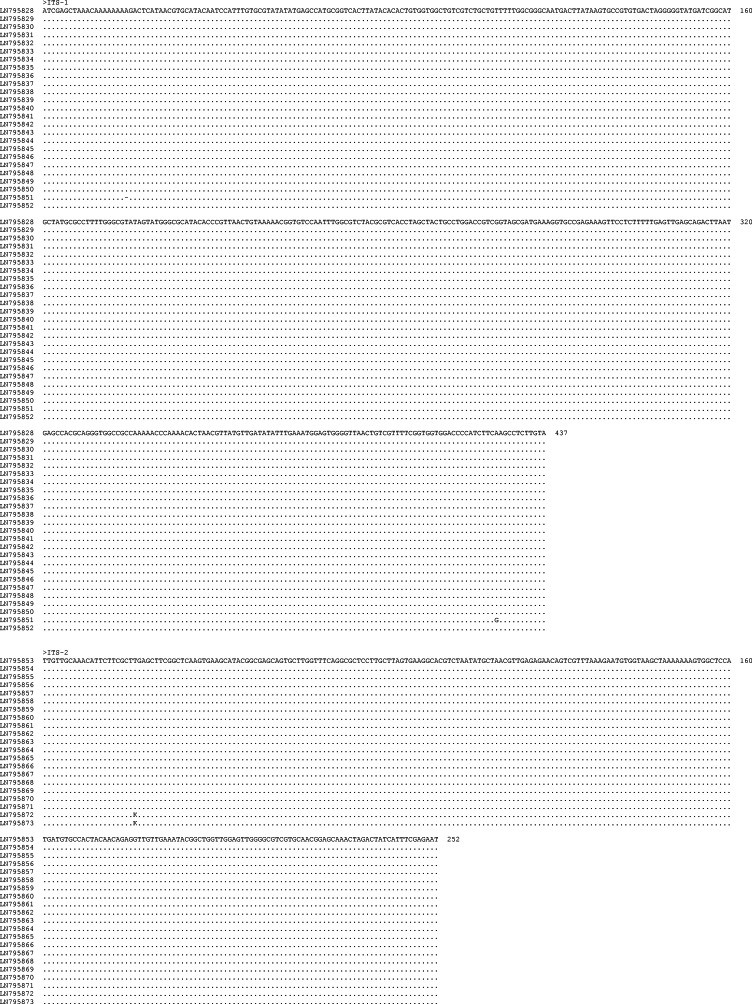
Alignment of the sequences of the ITS-1 and ITS-2 regions of *Terranova* larval type II of Cannon, 1977c found in the present study. The left column indicates the GenBank accession number of specimens. Numbers to the right of alignment indicate the alignment position. Polymorphic sites were designated using IUPAC codes.

The length of the ITS-1 was 437 bp except for two specimens which were 436 bp long. The difference in length was due to a gap at alignment position 20 in the latter specimens (Fig. 2). Also, sequence polymorphism was detected at alignment position 426 in one specimen (Fig. 2). Sequence variation in the ITS-1 among specimens was 0–0.4% and the G + C content was 47.6–47.9%. The length of the ITS-2 was 252 bp. Sequence polymorphism was detected at alignment position 22 in two specimens. Sequence variation among individuals was 0–0.4% and the G + C content was 46.4–46.8%. ITS-1 and ITS-2 sequences of *Terranova* larval type found in the present study were almost identical among all larvae.

## Discussion

Previously, Cannon (1977) described two distinct *Terranova* larval types, I and II, in Queensland waters which were later reported by other authors from other parts of Australia (e.g., Doupe et al., 2003; Moore et al., 2011). According to Cannon (1977), the main difference between larval types I and II is the ratio of intestinal caecum to ventriculus being 1:1 in the former and 2:1 in the latter morphotype. Based on the similarity in the ratio of intestinal caecum to ventriculus and considering the geographical location of larvae and matching it with presence of adult nematodes, he suggested *Terranova* larval type I in his study could be *Terranova chiloscyiti* and *Terranova* larval type II could be *T. galeocerdonis* or *T. scoliodontis.* Although some species within *Pseudoterranova* (e.g., *P. cattani*) have the same ratio of intestinal caecum to ventriculus and although *Pulchrascaris* has been reported from the same general location (Table 3), the possibility of these larvae being *Pulchrascaris* spp. or *Pseudoterranova* spp. was not discussed in Cannon’s work. In addition, assigning larval type to adults based on the ratio of intestinal caecum to ventriculus has been considered to be unreliable. Huizinga (1967) showed that the length of the intestinal caecum is shorter in smaller/younger larvae and increases as the larvae grow in length. This can affect the ratio of intestinal caecum to other organs, such as ventricular appendix or ventriculus. As a result the specific identity of *Terranova* larval types remains unknown. For the same reasons, despite of morphological resemblance between *Terranova* larval type in the present study and those described by Cannon (1977) there is no certainty that they are genetically similar or belong to the same species due to lack of comparable molecular data for Cannon’s specimens.

In an attempt to specifically identify *Terranova* larval type in the present study, we genetically characterised all *Terranova* larval type found in the present study from broad geographical region as well as a broad variety of fish species, based on their ITS-1 and ITS-2 sequences followed by phylogenetic analyses.

The nucleotide variation within *Terranova* larval type in the present study was very low (0–0.4% for both ITS-1 and ITS-2), and was within the range for nucleotide variation (0–0.2% and 0–0.4% for ITS-1 and ITS-2 respectively) calculated for members of the same species in the family Anisakidae (Shamsi et al., 2009b). This suggests they all should be the same genotype/species.

**Figure 3:**
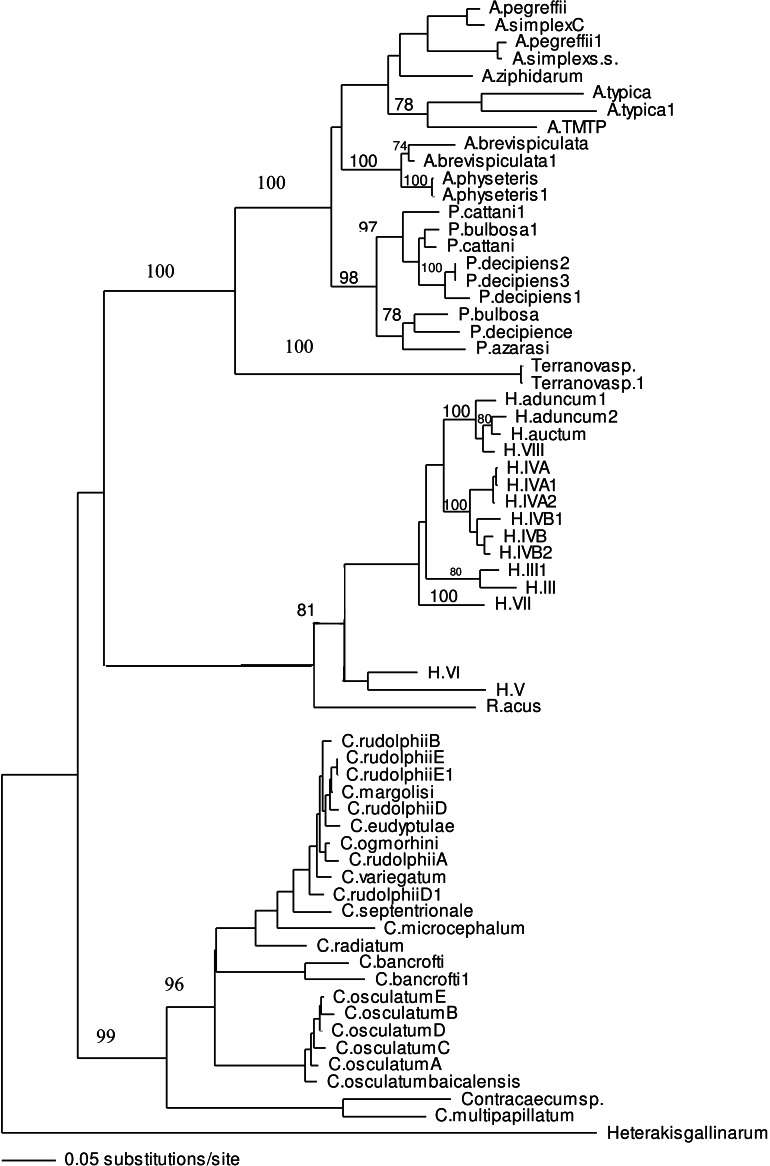
Phylogenetic analysis of the combined ITS-1 and ITS-2 sequence data for members of the Anisakidae with *Heterakis gallinarum* as outgroup, using the neighbour-joining method. Bootstrap support values are indicated. See Table 1 for detailed abbreviations. Note that Terranovasp and Terranovasp1 both belong to the same taxon and only different in polymorphic sites as shown in Fig. 2. They are representative of 93 *Terranova* larval type examined in the present study.

To reveal the specific identity of the *Terranova* larval type found in the present study comparable ITS sequences from well identified adults must be available. To date, there is no such sequence in the GenBank database. Among reliably identified species whose ITS-1 and ITS-2 sequences were available in the GenBank database, there was no identical or highly similar sequence to ITS-1 and ITS-2 sequences found in the present study. Alignment of ITS-1 and ITS-2 sequences of *Terranova* larval type in the present study with those available in GenBank database did not result in finding identical or highly similar sequences. Although the closest ITS sequences in the GenBank database belonged to *Pseudoterranova azarasi, P. bulbosa, P. cattani* and *P. decipiens sensu strict* the nucleotide difference between ITS sequences of the larvae in the present study and those of *Pseudoterranova* spp. in the GenBank was too great (38.9–39.8% and 46.7–48.4% for ITS-1 and ITS-2, respectively) to be considered within the genus *Pseudoterranova* (Table 4). The distinction between *Terranova* larval type in the present study and *Pseudoterranova* spp. was also supported by phylogenetic analyses (Fig. 3).

ITS-1 and ITS-2 sequences of well identified closely related taxa were selected to build the phylogenetic tree to investigate the association of larvae in the present study with other taxa within family Anisakidae. Both neighbour joining and maximum parsimony (the latter is not shown) trees had similar profile and grouping of taxa were the same among both trees. In the neighbour joining phylogenetic tree (Fig. 3), *Terranova* larval type found in the present study were resolved as a distinct clade with strong bootstrap support of 100%. None of the anisakid species (*Pseudoterranova* spp., *Anisakis* spp. and *Contracaecum* spp. becoming adult in marine mammals) with similar morphology to *Terranova* spp. (i.e., having excretory pore opened at the base of the labia) were grouped in the same clade as *Terranova* larval type found in the present study. Closely related species becoming adult in teleost fishes (*Hysterothylacium* spp. and *Raphidascaris acus*) were also included in the phylogentic tree, although the excretory system in this group has a different feature to *Terranova* spp. These anisakids also resolved as a distinct clade to *Terranova* larval type.

In both phylogenetic trees produced in the present study based on the combined ITS-1 and ITS-2 sequences, the *Terranova* larval type was resolved separately from *Pseudoterranova* spp. suggesting they do not belong to the genus *Pseudoterranova.*

As reviewed in the Introduction, Australian *Terranova* larval types could potentially be larval stages of *Pseudoterranova* spp., *Terranova* spp., or *Pulchrascaris* spp. Species associated with these genera have been listed in Table 3. Comparison of ITS sequence of *Terranova* larval type found in the present study with those of *Pseudoterranova* spp. available in GenBank (Table 4) shows a considerable nucleotide difference of 38.9–39.8% in both ITS-1 and ITS-2 regions. This is greater than nucleotide difference found for distinct species within a genus of family Anisakidae (Shamsi et al., 2009b) suggesting *Terranova* larval type in the present study does not belong to the genus *Pseudoterranova.*

**Table 4:** Pairwise comparisons of the nucleotide differences (%) in the consensus sequences of ITS-1 and ITS-2 between *Terranova* larval type found in the present study and *Pseudoterranova spp.* (the only taxa with closest ITS sequence similarity available in GenBank database).

	*Terranova* larval type in the present study	
	ITS-1	ITS-2
*P. azarsi*	39.8	46.7
*P. bulbosa*	38.9	48.4
*P. cattani*	39.3	47.2
*P. decipiens sensu tricto*	39.0	48.3

**Figure 4:**
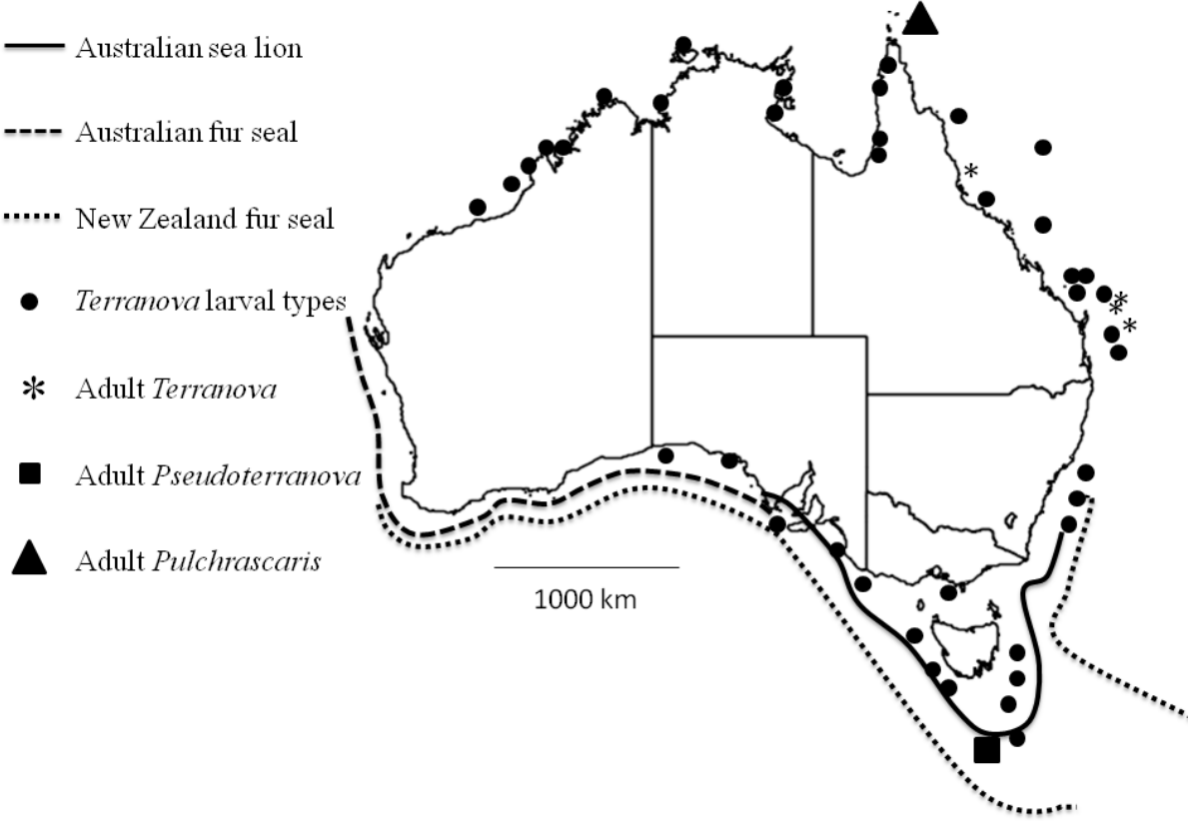
Map shows reported cases of *Terranova* larval types (circles), Adult *Terranova* spp (asterisk), adult *Pseudoterranova* spp (square), adult *Pulchrascaris* (triangle), distribution of Australian sea lion (solid line), Australian fur seal (square dots) and New Zealand fur seal (round dot).

To date, four species of *Terranova* have been reported from Australian sharks, *T. galeocerdonis, T. ginglymostomae, T. pristis* and *T. scoliodontis* (Bruce & Cannon, 1990). In addition, *T. crocodyli* was found in Australian crocodiles (Sprent, 1979). They all have a similar relationship between length of the intestinal caecum and ventriculus to the *Terranova* larval type in the present study. *Pulchrascaris* is a small genus in terms of number of species under family Anisakidae. Like members of the genus *Terranova, Pulchrascaris* spp. become adult in elasmobranches. There is intra/inter specific variation in the ratio of the intestinal caecum to ventriculus of *Pulchrascaris* spp. (Bruce & Cannon, 1990). Since there is no ITS sequences available for *Terranova* spp. or *Pulchrascaris* spp. in the GenBank database, the specific identity of the *Terranova* larval type found in the present study remains unknown and we are not able to associate these larvae to any *Terranova* spp. or *Pulchrascaris* spp. however, the present study, particularly the ITS sequence data, provides the essential information for future studies when the adult form is found and characterised.

This is the first report of a *Terranova* larval type from *Abudefduf whitleyi, Carangoides fulvoguttatus, Caranx ignobilis, C. melampygus, Chaetodon flavirostris, Lutjanus argentimaculatus, L. bohar, Pristipomoides multidens, Scomber australasicus.* Some of these fish, such as Australian mackerel (*Scomber australasicus)* are popular edible fish. Infection of those fish species that are not edible is also very important due to their role in the survival and transmission of *Terranova* larval type in the ecosystem.

Although the present study could not specifically identify the *Terranova* larval type in Australian waters, it could rule out the possibility of them being *Pseudoterranova* larvae which would have different implications for seafood and consumers’ safety and policy development in the country. It should be emphasized that it is very likely that *Pseudoterranova* larvae exist in Australian waters, infect some fish and await discovery. Their definitive hosts, Australian sea lion, Australian fur seal and New Zealand fur seal are found in southern coast of Australia (Fig. 4) and have been found to be infected with *Pseudoterranova decipiens E* (Bullini et al., 1997). However, given that in Australian waters the diversity of elasmobranch species is considerably higher (approximately 200 species, www.fishbase.net) than that of marine mammals (3 species of seals) our suggestion is that *Terranova* larval type in Australian waters is more likely to be a *Terranova* or a *Pulchrascaris*. To date there is no evidence that larval stage of *Terranova* or *Pulchrascaris* can cause infection in humans.

## Additional Information and Declarations

### DNA Deposition

The following information was supplied regarding the deposition of DNA sequences: Materials examined morphologically were deposited in South Australian Natural History Museum. The isolate numbers are as below: 301-1, 304-18, 304-2, 316-2, 324-4, 333-10, 336-4, 336-6, 340-4 and 341-3. Nucleotide sequence data reported in this paper are available in the GenBank database under the accession numbers LN795828– LN795873.

### Data Availability

The following information was supplied regarding data availability: The research in this article did not generate any raw data.
